# A Decade of Artificial Intelligence in Stroke Care (2015–2025): Trends, Clinical Translation, and the Precision Medicine Frontier—A Narrative Review

**DOI:** 10.3390/jpm16040218

**Published:** 2026-04-16

**Authors:** Mian Urfy, Mariam Tariq Mir

**Affiliations:** 1 Advocate Health, Chicago Medical School, Rosalind Franklin University, Chicago, IL 60064, USA; 2 Advocate Health, Chicago, IL 60068, USA; mariam.mir@aah.org

**Keywords:** artificial intelligence, machine learning, stroke, precision medicine, deep learning, neuroimaging, rehabilitation, atrial fibrillation, large language models, federated learning

## Abstract

**Background/Objectives:** Stroke generates 157 million disability-adjusted life-years (DALYs) annually, making it the leading neurological cause of global disease burden. Artificial intelligence (AI) and machine learning (ML) have emerged as transformative technologies across the stroke care continuum. This narrative review maps the trajectory of AI in stroke medicine over the decade from 2015 to 2025. **Methods:** We conducted a narrative review with a structured, pre-specified search strategy across eight pre-specified thematic clusters using PubMed/MEDLINE (January 2015–December 2025), identifying 8549 records and including 1335 studies after screening. Inclusion criteria encompassed primary research articles, systematic reviews, meta-analyses, and RCTs reporting quantitative performance metrics or clinical outcome data for AI/ML in stroke. **Results:** Stroke imaging AI is the most commercially mature domain, with over 30 FDA-cleared tools. Automated ASPECTS scoring reduced radiologist reading time by 74.8% (AUC 84.97%; 95% CI: 83.1–86.8%). The only triage AI RCT demonstrated an 11.2 min reduction in door-to-groin time without significant improvement in 90-day functional independence (OR 1.3, 95% CI 0.42–4.0). Brain–computer interface rehabilitation showed significant upper limb recovery in a 17-center RCT (FMA-UE mean difference +3.35 points, 95% CI 1.05–5.65; *p* = 0.0045). AF detection AI is FDA-cleared and RCT-validated. LLMs and federated learning are pre-regulatory but growing exponentially. **Conclusions:** AI in stroke has achieved diagnostic maturity but therapeutic immaturity. Bridging algorithmic performance to patient outcomes, addressing equity gaps, and building the economic evidence base for scalable deployment are the defining challenges of the next decade.

## 1. Introduction

Stroke remains one of the most devastating diseases affecting humanity. The Global Burden of Disease (GBD) Study 2021 reported that stroke generated 160.4 million disability-adjusted life-years (DALYs) globally, ranking it the leading neurological cause of DALYs worldwide [1]. Updated GBD 2023 estimates confirm stroke accounts for 157 million DALYs annually, second only to ischemic heart disease among non-communicable diseases [2]. Beyond mortality, stroke is the leading cause of serious long-term disability in adults, a burden that falls disproportionately on low- and middle-income countries (LMICs), where over 80% of stroke events now occur [3].

Despite advances in reperfusion therapy—from intravenous thrombolysis to mechanical thrombectomy—outcomes remain highly variable and difficult to predict at the individual level. Stroke is not a single disease but a heterogeneous syndrome encompassing ischemic and hemorrhagic subtypes, multiple etiologies, and widely varying severity, recovery trajectories, and post-stroke sequelae. Meaningful progress requires individualized, data-driven decision-making: in short, precision medicine.

AI and ML have emerged as transformative technologies in this context. Over 30 AI tools have received FDA clearance or CE marking and are deployed in thousands of hospitals worldwide.

However, this growth has been uneven. Diagnostic imaging AI has achieved mature clinical deployment and RCT-level validation. By contrast, AI for outcome prediction, rehabilitation, and risk stratification remains largely confined to single-center research. The only published RCT of an AI triage tool demonstrated reductions in door-to-treatment time but no significant improvement in functional independence at 90 days [4]. AI models also consistently underperform in underrepresented populations, raising urgent equity concerns. The economic evidence base for implementation remains almost entirely model based.

Against this background, this review synthesizes evidence on AI and ML in stroke medicine from 2015 to 2025, maps the field across eight thematic clusters, evaluates clinical translation and regulatory status, and identifies critical unmet needs for precision stroke care.

## 2. Materials and Methods

### 2.1. Study Design

This article presents a narrative review with a structured, pre-specified search strategy, conducted in accordance with the Scale for the Assessment of Narrative Review Articles (SANRA) framework [5]. The search strategy, eligibility criteria, and thematic framework were defined before data extraction commenced. Independent dual screening, inter-rater reliability assessment, and GRADE-level evidence synthesis were not performed, consistent with the narrative review design. The PRISMA 2020 flow diagram is included for transparency of the search and selection process only and does not imply systematic review methodology.

### 2.2. Literature Search Strategy

**Figure 1:**
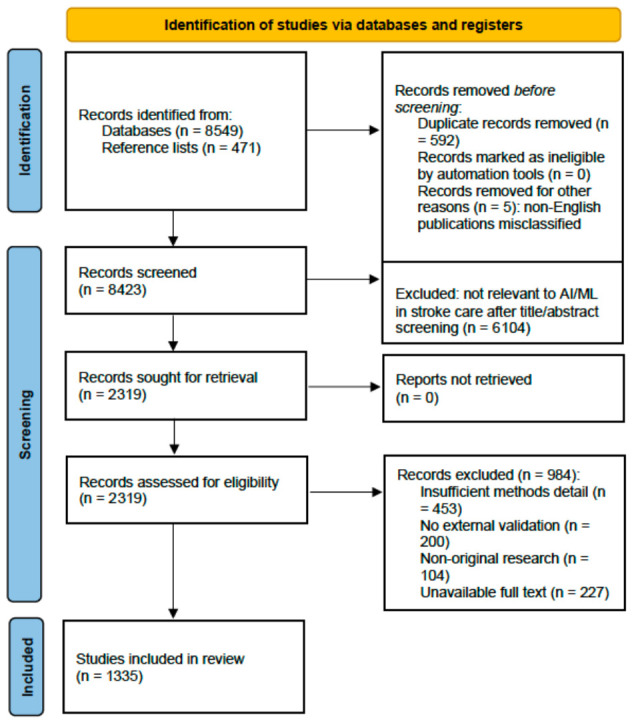
PRISMA-style search and selection flow diagram. Records were identified across eight thematic cluster searches in PubMed/MEDLINE (January 2015–December 2025) plus manual reference list screening. After title/abstract screening, full-text review, and application of eligibility criteria, 1335 studies were included in the narrative synthesis. Excluded records are shown with reasons at each stage. Some studies contribute to more than one thematic cluster; the total n per cluster therefore sums to more than 1335. Of the 8428 records retained after duplicate removal, 5 were subsequently identified as non-English publications that had been misclassified during automated deduplication; these were excluded prior to title/abstract screening, yielding 8423 records screened. PRISMA = Preferred Reporting Items for Systematic Reviews and Meta-Analyses; AF = atrial fibrillation; BCI = brain–computer interface; FL = federated learning; LLM = large language model; NLP = natural language processing.

Searches were conducted in PubMed/MEDLINE, covering publications from 1 January 2015 to 31 December 2025. Eight thematic clusters were searched independently using MeSH terms and free-text keywords (Table 1). All searches were limited to English-language publications. The complete search and selection process is presented in Figure 1.

### 2.3. Eligibility Criteria

**Inclusion:** Primary research articles, systematic reviews, meta-analyses, and RCTs reporting quantitative performance metrics (AUC, sensitivity, specificity, effect size, 95% confidence intervals) or clinical outcome data for AI/ML in stroke; studies involving human participants or validated clinical datasets; published 2015–2025 in English; indexed in PubMed/MEDLINE.

**Exclusion:** Conference abstracts without peer-reviewed full text; animal-only studies; studies not specifically addressing stroke; studies with no quantitative outcome reporting; letters and editorials without original data; duplicate publications from the same cohort without additional analysis.

### 2.4. Data Extraction and Quality Assessment

**Figure 2:**
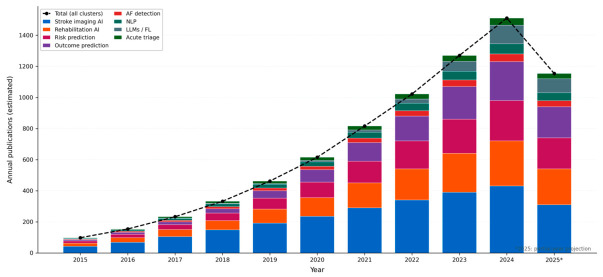
Estimated annual PubMed-indexed publications on AI in stroke by thematic cluster, 2015–2025. Stacked bars represent eight thematic clusters; the dashed black line shows total annual publications across all clusters. The explosive growth of LLMs and federated learning (gray) from near-zero before 2020 is particularly notable. * 2025 values are partial-year projections based on annualized rates from searches conducted February 2026. AF = atrial fibrillation; FL = federated learning; LLM = large language model; NLP = natural language processing.

For each included study, the following data were extracted: first author, publication year, journal, study design, population and sample size, AI/ML method used, primary outcome, performance metrics with 95% CIs where reported, clinical setting, and regulatory status. Annual publication volume per cluster was estimated from PubMed search totals to construct Figure 2. Study quality was assessed using PROBAST for prognostic models, QUADAS-2 for diagnostic accuracy studies, and Cochrane RoB 2.0 for RCTs. Given the narrative design, formal statistical pooling was not performed.

### 2.5. Regulatory Status Classification

**Figure 3:**
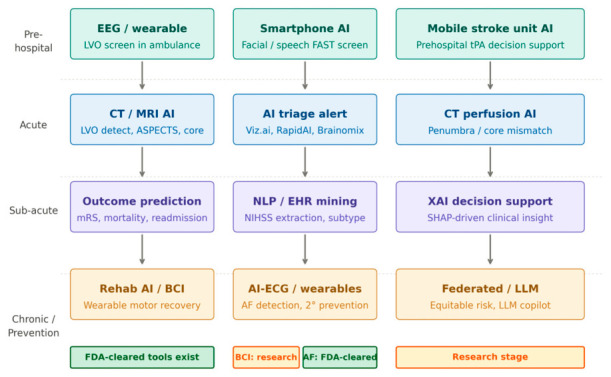
AI applications across the stroke care continuum by clinical phase. Each row corresponds to one clinical phase, and each box represents a distinct AI application domain. Box color reflects phase: teal = prehospital; blue = acute; purple = sub-acute; amber = chronic/prevention. Green status badges = FDA-cleared or CE-marked; amber badges = research stage. In the chronic/prevention row, BCI rehabilitation remains research-stage while AF wearable devices (Apple Watch, AliveCor KardiaMobile, Tempus ECG-AF) carry independent FDA clearances for AF detection, with stroke-prevention relevance deriving from the AF–anticoagulation–cardioembolic stroke pathway. BCI = brain–computer interface; FAST = Face Arm Speech Time; LVO = large vessel occlusion; XAI = explainable AI; LLM = large language model; AF = atrial fibrillation; NLP = natural language processing; EHR = electronic health record; SHAP = SHapley Additive exPlanations.

Each AI tool was classified as: (1) FDA-cleared (510(k) or De Novo); (2) CE-marked (EU MDR/MDD); (3) research-stage; or (4) pre-regulatory (Figure 3, Table 2). AF detection devices (Apple Watch, AliveCor KardiaMobile, Tempus ECG-AF) carry independent clearances for AF detection as a cardiac arrhythmia; their stroke-prevention relevance derives from the established AF–anticoagulation–cardioembolic stroke pathway.

## 3. Results

### 3.1. Publication Landscape: A Decade of Exponential Growth

**Figure 4:**
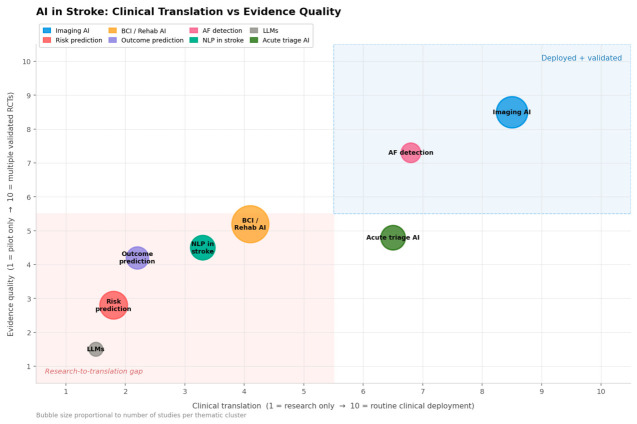
Evidence maturity matrix for AI in stroke (2015–2025). The x-axis represents clinical translation (1 = research only; 10 = widespread routine deployment). The y-axis represents evidence quality (1 = pilot studies; 10 = multiple validated RCTs). Bubble size reflects estimated cumulative publication volume. Only stroke imaging AI and AF detection occupy the deployed-and-validated quadrant (top-right). The research-to-translation gap quadrant (bottom-left) contains outcome prediction, risk prediction, NLP, and LLMs/FL. Dashed lines indicate quadrant boundaries. AF = atrial fibrillation; BCI = brain–computer interface; FL = federated learning; LLM = large language model; NLP = natural language processing.

Across all eight thematic clusters, estimated annual PubMed-indexed publications grew from approximately 98 per year in 2015 to over 1500 per year by 2024—a greater-than-15-fold expansion (Figure 2). Stroke imaging AI dominated throughout (~2550 cumulative papers, 33% of total output). The most striking recent growth belongs to LLMs and federated learning, which generated near-zero publications before 2020 but exceeded 150 annually by 2025. Outcome prediction demonstrated the steepest relative growth: approximately 17-fold from 49 papers (2015–2018) to 829 papers (2022–2025). Figure 1 illustrates how 8549 records identified across cluster searches were screened and refined to 1335 included studies. Figure 3 maps applications across the clinical continuum, and Figure 4 positions each cluster by evidence maturity.

### 3.2. Stroke Imaging AI: The Mature Frontier

Deep learning for stroke neuroimaging represents the largest and most commercially mature cluster (~2550 papers). Three core applications have dominated: LVO detection on CT angiography (CTA), automated ASPECTS scoring on NCCT, and CT perfusion (CTP) analysis [3,6].

For LVO detection, CNN-based algorithms achieve sensitivities of 90–98% in clinical deployment. Viz.ai—the first AI stroke tool to receive FDA De Novo clearance (2018)—operates across 1700+ hospitals in 100+ countries. Le et al. [6] demonstrated that ML-enabled LVO detection reduced DIDO time by a median of 77–106 min (*p* < 0.001) in a multicenter study (n = 115).

For automated ASPECTS scoring, Wei et al. [7] validated a deep learning system across 1987 NCCT scans from four centers, achieving an AUC of 84.97% (95% CI: 83.1–86.8%) and ICC of 0.84. Combining AI with physician review improved the AUC from 67.43% to 89.76% while reducing reading time by 74.8% (130.6 ± 61.3 s to 33.3 ± 8.3 s; *p* < 0.001). A prospective application study (n = 13,399) confirmed 94% of processed scans were used by clinicians. Independent validation by Ha et al. [8] confirmed higher inter-software than inter-expert agreement in the early treatment window (ICC 0.751 vs. 0.434; *p* = 0.018).

CT perfusion AI has achieved the greatest clinical impact. RAPID software version 4 underpinned the DEFUSE 3 and EXTEND RCTs, establishing the evidence base for extending the treatment window to 16–24 h. Cao et al. [9] compared istroke against RAPID across 326 patients, finding strong infarct core correlation (ρ = 0.68, *p* < 0.001) and substantial large-core agreement (κ = 0.73, *p* < 0.001). However, 13% of automated RAPID maps proved unreliable due to motion artifact [10]. A deep neural network predicting final infarct from DWI alone achieved an AUC of 0.91 (95% CI: 0.87–0.95) and a volume correlation ρ = 0.73 [11].

### 3.3. Acute Triage AI: Workflow Gains, Outcome Evidence Pending

The Martinez-Gutierrez et al. stepped-wedge cluster RCT (2023, *JAMA Neurology*) [4] demonstrated AI-enabled LVO detection reduced door-to-groin puncture time by 11.2 min and CT-to-thrombectomy start by 9.8 min across four stroke centers. Viz.ai real-world data confirmed 96% sensitivity at a median specialist notification time under 6 min. Le et al. [6] corroborated a 77–106 min DIDO reduction following AI implementation.

Critically, the Martinez-Gutierrez RCT found no statistically significant improvement in 90-day functional independence (OR 1.3, 95% CI 0.42–4.0) [4]. This disconnect—AI demonstrably saves time but has not yet demonstrably saved function—is the central unresolved challenge in triage AI and the most urgent priority for future trial design.

### 3.4. Outcome Prediction: High Performance, Limited Translation

ML for stroke prognosis expanded approximately 17-fold over the decade, with algorithms evolving from SVMs (2015–2018) to multimodal fusion deep learning since 2022. Liu et al. [12] achieved an AUC of 0.91 (95% CI: 0.89–0.93) using multimodal NCCT–clinical data fusion across 1335 patients from six datasets. Herzog et al. [13] showed deep learning outperformed five experienced neurologists for thrombectomy outcome prediction when imaging data were available. Kokkotis et al. [14] and Oei et al. [15] demonstrated SHAP-based identification of actionable modifiable risk factors.

For stroke subtype classification, Chen et al. [16] trained nine ML models on the China National Stroke Registry (n = 5213), achieving an AUC of 0.932 (95% CI: 0.921–0.943) for small vessel occlusion, with 45.7% of undetermined-etiology patients identified as likely cardioembolic. For post-stroke sequelae prediction, Liu et al. [17] confirmed ML can predict cognitive impairment, motor dysfunction, aphasia, depression, fatigue, and organ disease across the full post-stroke continuum. For recurrence, Fan et al. [18] achieved 95% accuracy and an AUC of 0.95 (95% CI: 0.93–0.97) using a multimodal model (n = 634). A key limitation is near-absent external validation, and Hong et al. [19] confirmed worse discrimination in Black versus White individuals (C-index 0.64–0.69 vs. 0.76).

### 3.5. AF Detection AI and Precision Secondary Prevention

AI-based AF detection directly addresses the cryptogenic stroke paradigm. Aschbacher et al. [20] demonstrated deep learning on raw PPG waveforms achieved an AUC of 0.983 (95% CI: 0.971–0.995). The Apple Heart Study (n = 419,297) validated smartwatch-based screening at population scale. The EQUAL Trial (2025) found AF detection rates of 9.6% versus 2.3% (HR 4.40, 95% CI 2.10–9.22; *p* = 0.001). Karakasis et al. [21] confirmed that AI-powered ECG screening, wearable PPG, and AI-driven anticoagulation decision support collectively constitute a precision prevention pipeline (Table 2).

### 3.6. Rehabilitation AI and Brain–Computer Interfaces

Wang et al. [22] conducted the largest BCI rehabilitation RCT to date (17 centers, n = 296), demonstrating BCI motor imagery training significantly improved upper limb function (FMA-UE mean difference 3.35 points, 95% CI 1.05–5.65; *p* = 0.0045). Liu et al. [23] (n = 60) confirmed BCI-enhanced upper limb improvement (+8.0 points, *p* < 0.001) alongside significant cognitive gains. The Cochrane review of electromechanical-assisted gait training (62 RCTs, n = 2440) found these interventions doubled the odds of independent walking (OR 2.01, 95% CI 1.43–2.83; NNT = 8). AI-enabled multimodal post-stroke monitoring demonstrated a 42% reduction in fall-related injuries and net savings of $15,311 per participant over 24 months [24]. Alt Murphy et al. [25] identified steep learning curves and limited sustained real-world use as major barriers to uptake.

### 3.7. NLP, LLMs, and Emerging Technologies

NLP in stroke has transformed from rule-based systems to transformer models. Fernandes et al. [26] achieved NIHSS extraction from clinical notes with a Spearman ρ = 0.96 on external MIMIC validation. Dymm and Goldenholz [27] demonstrated structured CARDS prompting achieved 100% guideline adherence for thrombolysis decisions using GPT-4o. Jiang et al. [28] found recent LLMs provide medically safe responses to neurointerventional patient queries. Engelhard et al. [29] showed parity-constrained neural networks partially improve inter-group fairness in stroke risk prediction, though at a cost to intra-group calibration. However, 1055 publicly accessible customized health GPTs identified by Chu et al. [30] carried no FDA, EU MDR, or TGA approval, highlighting a critical governance gap.

Critical appraisal of LLMs in stroke requires moving beyond benchmark performance to address fundamental safety and deployment barriers. Despite impressive controlled-condition results, LLMs remain entirely pre-clinical in stroke: no prospective trial of LLM-assisted stroke decision support has been published, and no LLM has received FDA clearance as a Software as a Medical Device (SaMD) for stroke indications. Three categories of concern are particularly relevant. First, hallucination: in a systematic analysis of vision-language models in diagnostic imaging, Dutta et al. [31] reported factual errors in approximately 22% of AI-generated clinical reports despite high NLP benchmark scores; Su et al. [32] in a scoping review of LLMs across 95 medical diagnostic studies confirmed model hallucination as a critical barrier to clinical adoption. In stroke, where a hallucinatory error in thrombolysis eligibility assessment could be immediately life-threatening, these rates are clinically unacceptable without robust human oversight frameworks. Second, prompt sensitivity: LLM outputs are highly sensitive to prompt formulation, producing meaningfully different recommendations from minor rewording—incompatible with the reproducibility demands of time-critical stroke triage. The 100% guideline adherence of structured CARDS prompting [27] applies to highly constrained pre-validated architectures that do not reflect real emergency department variability. Third, absence of causal reasoning: current LLMs are statistical pattern-matchers without mechanistic clinical reasoning. Stroke treatment requires integration of imaging, time windows, contraindications, and patient values in a causally structured framework that language models cannot yet replicate. These limitations do not negate LLM potential as augmentation tools for documentation and patient education, but they confirm that the governance gap identified by Chu et al. [30] constitutes a patient safety concern, not merely a regulatory formality.

## 4. Discussion

### 4.1. The Diagnostic–Therapeutic Divide

The central finding of this review, made visually explicit by Figure 4, is a striking asymmetry: AI in stroke has achieved diagnostic maturity but therapeutic immaturity. Over 30 FDA-cleared imaging and AF detection tools are deployed in clinical practice, supported by multicenter validation and, in the case of CT perfusion software, pivotal RCT evidence. By contrast, no dedicated stroke outcome prediction, rehabilitation AI, or risk stratification tool has received regulatory clearance or achieved widespread clinical adoption. We acknowledge that characterizing this as a ‘divide’ rests on limited prospective evidence, and that the absence of therapeutic AI RCTs may partially reflect publication lag, longer regulatory pathways for interventional tools, and the inherent complexity of powering trials for functional stroke outcomes—rather than a fundamental therapeutic inefficacy of AI. The absence of evidence of therapeutic benefit is not equivalent to evidence of absence of benefit, and adequately powered RCTs with 90-day mRS as the primary endpoint remain the essential next step.

This divide is not merely a regulatory artifact—it reflects a genuine asymmetry in evidence quality. Imaging AI has been validated against objective endpoints in well-defined populations at decision-critical time points. Prognostic AI must contend with far greater outcome heterogeneity, longer follow-up requirements, and near-total absence of external validation. A 2022 meta-analysis found pooled AUCs of only 0.81 for thrombectomy outcome models and no general superiority over existing clinical scores [33].

The sole RCT of AI triage [4] reinforces this from the opposite direction. Viz.ai reduced notification time by 52 min and DIDO time by 77–106 min [6]—demonstrably saving time. Yet functional independence at 90 days did not improve significantly. Future RCTs must be powered for 90-day mRS as the primary endpoint, not workflow time metrics, to provide the definitive answer the field requires.

### 4.2. The Equity Gap: AI That Works Better for Some

A consistent and troubling pattern across this review is the underperformance of AI models in underrepresented populations. Hong et al. [19] demonstrated significantly worse discrimination in Black versus White individuals across all tested stroke risk models (C-index 0.64–0.69 vs. 0.76), with no improvement from novel ML approaches. Engelhard et al. [29] showed that parity-constrained models partially close inter-group discrimination gaps but necessarily sacrifice intra-group calibration—a trade-off with no universally correct answer, and one that must be decided contextually, with clinician and patient input.

The structural cause is clear: most stroke AI training datasets originate from high-income country academic centers, predominantly East Asian and European populations, while stroke burden is highest in sub-Saharan Africa and South Asia. Federated learning represents the most technically promising pathway, enabling privacy-preserving model training across geographically diverse institutions. Achieving genuinely global equity in stroke AI will require extending federated architectures to stroke registries in Nigeria, India, Brazil, and China—countries that bear the majority of global stroke burden but contribute a minority of current training data.

A more precise analysis of equity failures in stroke AI requires distinguishing three mechanistically distinct sources of bias, each demanding different mitigation strategies. First, dataset shift (covariate shift) arises when the input feature distribution of the training population does not match the target deployment population—for example, models trained on East Asian and European stroke cohorts underperforming in sub-Saharan African patients due to differences in stroke subtype prevalence, imaging protocols, and vascular risk factor profiles. Federated learning across geographically diverse registries is the primary technical response. Second, label bias occurs when the ground truth labels used for training reflect historical patterns of inequitable clinical decision-making—for example, systematic underdiagnosis or differential treatment of minority patients whose outcomes are then encoded as training targets. The finding by Hong et al. [19] that novel ML approaches failed to improve discrimination in Black individuals (C-index 0.64–0.69) beyond that of traditional models, despite superior overall performance, is consistent with label-level bias that fairness-constrained training and label auditing must address. Third, healthcare system structural bias arises from differential access to specialist neuroimaging, acute reperfusion therapy, and documented EHR data across socioeconomic and geographic strata—meaning that AI models trained on resource-rich center data may fail in lower-resourced settings not because of algorithmic deficiencies but because the underlying care received differs. This is a structural inequity that requires health system-level intervention beyond algorithmic correction. Current stroke AI equity research, including the Engelhard et al. [29] parity-constrained work, primarily addresses label bias through post hoc fairness constraints; dataset shift and system-level structural bias remain largely unaddressed in published stroke AI literature.

### 4.3. Post-Stroke Sequelae: A Neglected Precision Target

A significant gap in the current literature is the inadequate coverage of post-stroke sequelae prediction. Approximately 30% of stroke survivors develop post-stroke depression, 20% develop anxiety, and substantial proportions experience cognitive impairment, aphasia, fatigue, and organ-system complications. Liu et al. [17] confirmed that ML models for sequelae prediction are technically feasible and increasingly multimodal, but lag behind acute stroke AI in methodological rigor and clinical implementation.

From a precision medicine perspective, this is a high-value target. AI-based early identification of high-risk individuals for targeted psychological and rehabilitative intervention is precisely the kind of personalized care that precision stroke medicine encompasses. Oei et al. [15] demonstrated gradient-boosted trees with SHAP identified modifiable predictors of post-stroke adverse mental outcomes with 74.7% accuracy (95% CI: 72.1–77.3%). AI-enabled multimodal monitoring systems combining wearable sensors, computer vision, and voice analysis demonstrated a 94.8% fall detection sensitivity (95% CI: 92.6–96.3%) and 42% reduction in fall-related injuries in post-stroke community settings [24], suggesting that continuous AI-guided monitoring after discharge may dramatically reduce preventable harms.

### 4.4. Cost-Effectiveness and Implementation Economics

A striking omission in the current stroke AI evidence base is robust economic data. Brin and Tau [34] systematically reviewed cost-effectiveness of AI in radiology, identifying only ten eligible studies, all model-based, with no prospective real-world cost-effectiveness data. Despite over 30 FDA-cleared stroke AI tools deployed in thousands of hospitals, not a single published study has conducted a prospective economic evaluation of their real-world implementation costs and QALY gains. This is a critical barrier to scale-up: hospital systems and payers require health economic justification before committing to AI infrastructure investment.

In the rehabilitation domain, Xue et al. [24] provided one of the most compelling economic analyses to date, demonstrating net savings of $15,311 per participant over 24 months with AI-enabled multimodal post-stroke monitoring, alongside significant reductions in hospitalization and caregiver burden. Prospective economic evaluations embedded within future RCTs of stroke AI tools—imaging triage, prognostic models, and rehabilitation systems alike—should be considered mandatory components of the evidence generation strategy for the next decade.

### 4.5. Conservative Medical Management: An Underexplored Domain for AI

A critical gap in the current review is the near-absence of AI applications targeting conservative (non-reperfusion) ischemic stroke management. In clinical practice, the majority of ischemic stroke patients are managed with conservative therapies: antiplatelet agents (aspirin, clopidogrel, dual antiplatelet therapy), oral or intravenous anticoagulation for cardioembolic subtypes, neuroprotective agents such as edaravone, intensive blood pressure control, complication prevention (particularly stroke-associated pneumonia), and nutritional support. These domains collectively determine outcomes for the large proportion of patients who are either ineligible for or do not benefit from reperfusion therapy, yet they remain largely unaddressed in the AI literature reviewed here.

Regarding anticoagulation decision-making, Lu et al. [35] demonstrated that multilabel gradient-boosting ML models outperformed the CHA_2_DS_2_-VASc score for predicting stroke risk and significantly outperformed HAS-BLED for major bleeding risk in 9670 patients with non-valvular atrial fibrillation (AUC 0.709 vs. 0.522 for bleeding, NRI = 22.8%; *p* < 0.05), identifying hemoglobin level and renal function as additional risk features beyond conventional risk scores. This has direct relevance to secondary stroke prevention through optimized anticoagulant prescribing. For stroke-associated pneumonia (SAP)—the leading infectious complication after stroke and a major determinant of in-hospital mortality—Abujaber et al. [36] applied five ML models to 9840 patients from a national stroke registry, with an artificial neural network achieving an AUC of 0.94 and F1-score of 0.86, identifying stroke severity, dysphagia, and admission blood pressure as the dominant predictors. Tsai et al. [37] further showed that combining ML with NLP on unstructured clinical notes achieved an AUC of 0.840 (95% CI: 0.806–0.875) for SAP prediction, significantly outperforming all four conventional SAP risk scores. These findings demonstrate that AI can meaningfully improve the identification of patients at highest risk for the most common and preventable non-neurological complication of stroke.

For blood pressure management—a cornerstone of both acute ischemic stroke care and secondary prevention—Sharma et al. [38] reviewed emerging ML approaches for predicting individualized blood pressure treatment thresholds post-thrombectomy, noting that autoregulation-based ML targets represent a promising but pre-regulatory frontier. No prospective RCT of an AI-guided blood pressure protocol in acute ischemic stroke has yet been published, representing a significant evidence gap given the well-established impact of post-reperfusion hypertension on hemorrhagic transformation risk. The deliberate exclusion of these conservative management domains from the present review reflects the current state of the literature rather than a judgment about clinical importance. Future AI research must extend beyond the reperfusion window to address antiplatelet optimization, anticoagulant personalization, complication prediction, and supportive care—domains that determine outcomes for the majority of stroke patients worldwide.

### 4.6. Future Directions

**Priority 1: RCTs powered for patient-level functional outcomes.** The Martinez-Gutierrez RCT [4] was pivotal but underpowered for 90-day mRS as a primary endpoint. Future trials of AI triage, outcome prediction, and precision rehabilitation tools must define functional independence as the primary outcome and be adequately powered to detect clinically meaningful differences. Adaptive trial designs are particularly well-suited to this domain.

**Priority 2: External validation across diverse populations.** No prognostic AI model in this review demonstrated external validation across ethnically and geographically diverse populations. This must become a prerequisite for publication in high-impact journals and a condition for regulatory consideration, analogous to the TRIPOD-AI framework.

**Priority 3: Federated learning at global scale for equitable AI.** The TRUSTroke platform demonstrates federated learning feasibility in Europe but must be extended to LMIC stroke registries. Under the EU AI Act [39], AI medical devices face additional compliance requirements; international regulatory harmonization will be essential to avoid asymmetric burdens between high-income and low-income settings.

**Priority 4: Multimodal foundation models as the next architectural frontier.** Current stroke AI is largely single-modality and single-task. The next generation should integrate imaging, structured clinical data, genetic data, EHR free-text, and wearable sensor streams in unified multimodal foundation models. Convergence of vision-language models with federated training represents the highest-impact direction for the next five years.

**Priority 5: Governance frameworks for LLMs in clinical stroke care.** 1055 publicly accessible customized health GPTs identified by Chu et al. [30] operated without any regulatory approval. As LLMs become increasingly accessible to patients and clinicians for stroke-related queries, structured prompting frameworks such as CARDS [27] demonstrate safety can be substantially improved, but regulatory clarity on LLM-based clinical decision support as SaMD is urgently needed.

### 4.7. Limitations

This review has several important limitations. First, the narrative design with structured search elements means exhaustive retrieval was not performed and selection bias cannot be excluded. Second, searches were limited to PubMed/MEDLINE, which does not comprehensively index computer science or AI/ML conference proceedings; IEEE Xplore, ACM Digital Library, and arXiv host substantial foundational stroke AI methodology not captured here, meaning publication volume estimates in Figure 2 likely underestimate true output, particularly for imaging AI and deep learning clusters.

Third, the English-language restriction introduces systematic geographic bias, likely underrepresenting evidence from China, India, and sub-Saharan Africa. Fourth, the field is evolving at extraordinary pace; significant developments occurring during the peer review period will not be captured, particularly in the LLM and federated learning clusters. Fifth, publication volume estimates in Figure 2 are approximations from PubMed search counts rather than precise bibliometric counts, with overlapping terms between clusters meaning some articles may be counted multiple times, and 2025 values are partial-year projections.

Sixth, formal GRADE assessment of the body of evidence per clinical question was not performed, limiting the ability to make formal evidence-strength recommendations. Future systematic reviews with narrower scope should apply GRADE to specific clinical questions such as the impact of AI triage on 90-day functional outcomes. Finally, this review does not include a formal conflict-of-interest analysis of included studies; a number of highly cited papers were authored by researchers with financial relationships with the commercial entities whose tools they validated, representing a systemic limitation that independent replication should address.

## 5. Conclusions

The decade 2015–2025 transformed AI in stroke from a niche academic pursuit into a clinical reality, with billions of dollars in investment, dozens of regulatory-cleared tools, and an evidence base measured in thousands of publications. The dominant narrative is one of diagnostic triumph and translational challenge: algorithms that match or exceed expert performance in detecting stroke pathology, but a persistent inability to prove these tools improve what matters most—functional independence, survival, and quality of life.

For precision stroke medicine to be realized, five research priorities must be addressed: (1) RCTs powered for patient-level functional outcomes; (2) external validation in geographically and ethnically diverse populations; (3) federated learning expanded to LMIC stroke registries; (4) multimodal foundation models integrating imaging, clinical, and molecular data; and (5) governance frameworks for LLMs in clinical stroke decision support. The economic case for AI-enabled post-stroke monitoring is beginning to emerge [24], and the regulatory landscape is evolving with the EU AI Act [39] introducing new requirements for AI medical devices. The infrastructure is built. The algorithms are capable. The evidence and governance must now follow.

## Figures and Tables

**Table 1:** PubMed search strategy by thematic cluster.

Cluster	Core Search Terms	Date Range	Est. Papers
1. Stroke imaging AI	“deep learning” OR “CNN” AND “stroke” AND “CT” OR “MRI” OR “lesion segmentation”	2015–2025	~2550
2. Acute triage AI	“AI” AND “stroke” AND “triage” OR “large vessel occlusion”	2015–2025	~4400
3. Outcome prediction	“machine learning” AND “stroke” AND “outcome” OR “modified Rankin”	2015–2025	~1100
4. AF detection/subtype	“AI” AND “atrial fibrillation” AND “stroke” OR “ECG” OR “wearable”	2015–2025	~200
5. Rehabilitation AI	“BCI” OR “brain–computer interface” AND “stroke rehabilitation”	2015–2025	~1840
6. Risk prediction	“machine learning” AND “stroke” AND “risk prediction”	2015–2025	~1314
7. NLP in stroke	“natural language processing” AND “stroke” OR “EHR”	2015–2025	~245
8. LLMs/federated learning	“large language model” OR “federated learning” AND “stroke”	2023–2025	~300

Searches were conducted in PubMed/MEDLINE between January and February 2026. All searches were limited to English-language publications with no restriction on study design. Estimated paper counts are derived from PubMed total result counts and should be interpreted as approximate. AF = atrial fibrillation; BCI = brain–computer interface; CNN = convolutional neural network; EHR = electronic health record; LLM = large language model; NLP = natural language processing.

**Table 2:** FDA-cleared AI tools relevant to stroke care.

Tool	Company	FDA Pathway	Year	Application	Key Evidence
Viz LVO	Viz.ai	De Novo	2018	LVO detection on CTA; care coordination alert	First FDA-cleared stroke AI; 96% sensitivity; <6 min notification
RAPID (RapidAI)	iSchemaView	510(k)	2016–2020	CT perfusion—infarct core and penumbra	Used in DEFUSE 3 and EXTEND RCTs; 2500+ hospitals
Brainomix e-ASPECTS	Brainomix	510(k) + CE	2021	Automated ASPECTS on NCCT	CE-marked; higher inter-software than inter-expert agreement
Brainomix e-CTP	Brainomix	510(k) + CE	2022	CT perfusion analysis	CE-marked; compared favorably to RAPID
Qure.ai qER	Qure.ai	510(k)	2020–2023	ICH, midline shift, mass effect on CT	19 FDA clearances; AUC > 0.95 for ICH detection
Methinks	Methinks	510(k)	2022–2024	LVO on CTA and NCCT	98.2% sensitivity for CTA-based LVO
Apple Watch AF	Apple	De Novo	2018	AF detection via PPG/ECG	Apple Heart Study n = 419,297; EQUAL Trial 9.6% vs. 2.3%
AliveCor KardiaMobile	AliveCor	510(k)	2014+	Single-lead ECG AF detection	First consumer AI-ECG cleared for AF
Tempus ECG-AF	Tempus	510(k)	2023	12-lead ECG AF risk detection	AI-ECG AUC 0.87–0.90 for latent AF

AF = atrial fibrillation; CTA = CT angiography; ICH = intracerebral hemorrhage; LVO = large vessel occlusion; NCCT = non-contrast CT; PPG = photoplethysmography. AF tools carry clearances for AF detection as a cardiac arrhythmia; stroke-prevention relevance derives from the AF–anticoagulation–cardioembolic stroke pathway.

## Data Availability

No new data were created in this study.

## Abbreviations

The following abbreviations are used in this manuscript:

AI	Artificial Intelligence
AUC	Area Under the Receiver Operating Characteristic Curve
AF	Atrial Fibrillation
BCI	Brain–Computer Interface
CI	Confidence Interval
CNN	Convolutional Neural Network
CT	Computed Tomography
CTA	CT Angiography
CTP	CT Perfusion
DALY	Disability-Adjusted Life Year
DIDO	Door-In-Door-Out
DWI	Diffusion-Weighted Imaging
EHR	Electronic Health Record
FDA	US Food and Drug Administration
FMA-UE	Fugl-Meyer Assessment Upper Extremity
GBD	Global Burden of Disease
ICH	Intracerebral Hemorrhage
LMIC	Low- and Middle-Income Country
LLM	Large Language Model
LVO	Large Vessel Occlusion
ML	Machine Learning
mRS	Modified Rankin Scale
MRI	Magnetic Resonance Imaging
NCCT	Non-Contrast Computed Tomography
NIHSS	NIH Stroke Scale
NLP	Natural Language Processing
OR	Odds Ratio
PPG	Photoplethysmography
PRISMA	Preferred Reporting Items for Systematic Reviews and Meta-Analyses
QALY	Quality-Adjusted Life Year
RCT	Randomized Controlled Trial
SANRA	Scale for the Assessment of Narrative Review Articles
SaMD	Software as a Medical Device
HR	Hazard Ratio
ICC	Intraclass Correlation Coefficient
NNT	Number Needed to Treat
NRI	Net Reclassification Improvement
SHAP	SHapley Additive exPlanations
XAI	Explainable AI
FL	Federated Learning
SAP	Stroke-Associated Pneumonia
